# To: What's the best strategy for biomarker-guided antibiotic therapy in sepsis

**DOI:** 10.62675/2965-2774.20260185-2

**Published:** 2026-08-31

**Authors:** Vanessa Soares Lanziotti, Roberto Jabornisky, Saraswati Kache

**Affiliations:** 1 Universidade Federal do Rio de Janeiro Instituto de Puericultura e Pediatria Martagão Gesteira Research & Health Technologic Innovation Unit Rio de Janeiro RJ Brazil Research & Health Technologic Innovation Unit, Instituto de Puericultura e Pediatria Martagão Gesteira, Universidade Federal do Rio de Janeiro - Rio de Janeiro (RJ), Brazil.; 2 Universidad Nacional de Nordeste Department of Pediatrics Corrientes Argentina Department of Pediatrics, Universidad Nacional de Nordeste - Corrientes, Argentina.; 3 Lucile Packard Children's Hospital Department of Pediatrics Division of Critical Care Medicine California United States Division of Critical Care Medicine, Department of Pediatrics, Lucile Packard Children's Hospital, Stanford Medicine - California, United States.

We read with great interest the article published by Guerreiro et al. in Critical Care Science.^(1)^ The authors presented a concise and global outline of biomarker-guided antibiotic therapy in adult sepsis, focusing primarily on biomarkers with the strongest evidence at the bedside, procalcitonin (PCT) and C-reactive protein (CRP). However, because children are not small adults and the pediatric evidence base is considerably smaller, recommendations for biomarker use in pediatric sepsis may diverge from those in adults.

Over the last decade, the field of biomarker and immuno-profiling has exploded as we begin to understand the various forms of hyperinflammatory states, including specific pediatric sepsis phenotypes. Although these are powerful research tools, at the bedside CRP and PCT remain the main biomarkers used due to ease of availability in multi-resource settings.^(2)^

Although CRP has served as a biomarker in pediatric sepsis for several decades, its diagnostic utility is constrained by limited sensitivity and specificity, when used as a single measurement. However, when checked serially, the CRP trajectory proves to be beneficial. For example, pediatric patients at risk of poor outcomes due to sepsis can be identified by trending the CRP response to antibiotic therapy during the first 7 days of admission.^(3)^ Similar findings have been demonstrated in adult populations. This dynamic approach can guide clinical decision-making, particularly when the expected decline in CRP fails to occur or when a biphasic pattern emerges – an initial decline followed by a subsequent rise. Thus, when CRP can be trended longitudinally, it proves to be a useful biomarker both for therapeutic response and prognosis.

While Guerreiro et al.^(1)^ note PCT's value in guiding de-escalation in adult sepsis, evidence for this use in pediatrics remains weak. The BATCH trial (Biomarker-guided duration of Antibiotic Treatment in Children Hospitalized with confirmed or suspected bacterial infection) was a multicenter randomized controlled trial conducted in the United Kingdom that enrolled 1,949 pediatric patients. Results of this trial published in 2025 demonstrated that PCT-guided algorithms did not reduce intravenous antibiotic duration compared to standard care.^(4)^ The most recent Surviving Sepsis Campaign International Guidelines for pediatric sepsis, published in March 2026, similarly do not recommend routine PCT use to guide antibiotic de-escalation if effective antimicrobial stewardship programs are in place.^(5)^ However, dynamic PCT monitoring, like CRP, may still offer prognostic value: a 2025 study of 242 children with sepsis found that lower PCT clearance within the first 24 hours of antibiotic therapy was significantly associated with higher mortality.^(6)^ That said, PCT is more costly than CRP; its levels can be confounded by acute renal failure and corticosteroid use; and testing remains unavailable in many institutions worldwide.

However, the greatest challenge in pediatric sepsis remains *when* antibiotics should be initiated. Unfortunately, the latest Surviving Sepsis Campaign International Guidelines do not address this question. This gap is partially filled by the American Academy of Pediatrics (AAP) guidelines for febrile infants under 90 days of age, which incorporate biomarker-guided antibiotic initiation protocols. The AAP recommends antibiotic initiation in patients with the following diagnostics: a CRP ≥ 20mg/L identifies those at higher risk, and procalcitonin (> 0.5ng/mL) serves as an independent predictor of bacterial infection.^(7)^

Yet as every clinician recognizes, individual laboratory studies and their trajectories are only pieces of a larger puzzle; only when combined with the history and physical examination do they provide a holistic picture of the patient. As Djulbegovic et al.^(8)^ wisely noted, "evidence alone never speaks for itself or conveys the truth because it always requires interpretation". The AAP itself reminds us that "a number is not a decision". Much work remains in biomarker and sepsis research, but one principle holds across age groups: biomarkers do not replace clinical judgment and must always be interpreted within that context.
